# Comparative Analysis of Protist Communities in Oilsands Tailings Using Amplicon Sequencing and Metagenomics

**DOI:** 10.1111/1462-2920.70029

**Published:** 2025-01-10

**Authors:** Kristína Záhonová, Harpreet Kaur, Chantel C. Furgason, Angela V. Smirnova, Peter F. Dunfield, Joel B. Dacks

**Affiliations:** ^1^ Division of Infectious Diseases, Department of Medicine, and Department of Biological Sciences University of Alberta Edmonton Alberta Canada; ^2^ Institute of Parasitology, Biology Centre Czech Academy of Sciences České Budějovice Czech Republic; ^3^ Department of Parasitology, Faculty of Science Charles University Vestec Czech Republic; ^4^ Life Science Research Centre, Faculty of Science University of Ostrava Ostrava Czech Republic; ^5^ Department of Biological Sciences University of Calgary Calgary Alberta Canada

**Keywords:** amplicon, diversity, metagenome, mitochondrial genome, oilsands, protist

## Abstract

The Canadian province of Alberta contains substantial oilsands reservoirs, consisting of bitumen, clay and sand. Extracting oil involves separating bitumen from inorganic particles using hot water and chemical diluents, resulting in liquid tailings waste with ecotoxicologically significant compounds. Ongoing efforts aim to reclaim tailings‐affected areas, with protist colonisation serving as one assessment method of reclamation progress. Oilsands‐associated protist communities have mainly been evaluated using amplicon sequencing of the 18S rRNA V4 region; however, this barcode may overlook important protist groups. This study examined how community assessment methods between the V4 and V9 regions differ in representing protist diversity across four oilsands‐associated environments. The V9 barcode identified more operational taxonomical units (OTUs) for Discoba, Metamonada and Amoebozoa compared with the V4. A comparative shotgun metagenomics approach revealed few eukaryotic contigs but did recover a complete *Paramicrosporidia* mitochondrial genome, only the second publicly available from microsporidians. Both V4 and V9 markers were informative for assessing community diversity in oilsands‐associated environments and are most effective when combined for a comprehensive taxonomic estimate, particularly in anoxic environments.

## Graphical abstract

Protist diversity in oilsands‐associated environments has been used as a reclamation indicator, primarily assessed using the V4 barcode. This study indicates that the V9 barcode identifies previously undetected Amoebozoa, Discoba and Metamonada OTUs, particularly in anaerobic oilsands environments. Nevertheless, both barcodes exhibit similar relative patterns of community composition.

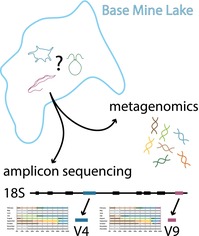

## 1 Introduction

Global energy consumption is projected to increase by 27% by the year 2040, driven by economic expansion in both developed and emerging nations (IEA 2021). With the decline in conventional oil sources, the demand for unconventional petroleum, such as oilsands, is rising (Hein 2006). Oilsands are a mixture of water, sand, and bitumen. Oilsands are spread worldwide including in Venezuela, Russia, the USA, and Canada, accounting for 24% of proven oil reserves in Venezuela (261.8 billion barrels) and Canada (166.3 billion barrels) (Hein 2017; Ersoy, Schaffer, and Ditzen 2019). The Athabasca oilsands, situated in Northern Alberta, represent the world's third‐largest oil reserve (Government of Alberta 2023). The recovery of bitumen, primarily by open‐pit mining, followed by extraction with hot caustic water and chemical diluents, has affected the natural environment via disturbance of land and fragmentation of wildlife habitat, river water usage, environmental contamination with naphthenic acids, and volatile hydrocarbon emissions (Brown and Ulrich 2015; Holden, Donahue, and Ulrich 2011; Jordaan 2012; Small et al. 2015).

Tailings ponds are large‐scale settling basins used for the collection of liquid waste byproducts of oilsands processing (Nix and Martin 1992). One of the reclamation strategies for ecological restoration of mined land is the formation of end‐pit lakes (EPLs) (Hrynyshyn 2012). In this process, a freshwater cap is added to a layer of tailings in a mined‐out pit. The tailings should consolidate over time and be sequestered, while the water cap provides an oxic environment for microbial biodegradation (Foght, Gieg, and Siddique 2017). Base mine lake (BML) is located 45 km north of Fort McMurray, Alberta, and is the first full‐scale pit lake of the oilsands industry in Canada. The 8 km^2^ area of BML contains 169 million L of fluid fine tailings and a water cap of approximately 71 million L of mixed freshwater and oilsands process water (Suncor Energy (Syncrude) Operating Inc. (SESOI) 2023). The water level in BML is maintained by pumping in fresh water from a reservoir (Beaver Creek Reservoir, or BCR) and/or pumping out to an extraction plant. BML displays a typical dimictic pattern of thermal stratification. Ice‐off occurs by April to early May, spring turnover from late May to mid‐June, stratification from mid‐June to early September, autumn turnover by early September and ice‐on mid‐November (Lawrence, Tedford, and Pieters 2015). With time, it is hoped that BML will develop into a functional ecosystem (White and Liber 2018). The proximity of BML to active tailings ponds and a freshwater reservoir provides an opportunity to compare the microbial communities in these respective water bodies and their ecological roles (Foght, Gieg, and Siddique 2017; Golby et al. 2012; Wu et al. 2020).

The microbial communities in tailings ponds have been previously studied to understand their roles in biodegradation processes. Monitoring community development is also a potential tool to assess environmental health (Aguilar et al. 2016; Holowenko, MacKinnon, and Fedorak 2001; Penner and Foght 2010; Ramos‐Padrón et al. 2011; Furgason et al. 2024). Biodiversity estimation with next‐generation sequencing and metagenomics of environmental DNA provides a high coverage estimate of community composition (Liddicoat et al. 2022; van der Heyde et al. 2020). However, the metagenomic approach is limited by a lower availability of eukaryotic genome databases as compared to prokaryotic ones, and horizontal gene transfer leading to inaccurate species identification (Hong, Mantilla‐Calderon, and Wang 2020; Tessler et al. 2017). It was previously shown that eukaryotic diversity estimated using metagenomic techniques was limited by high prokaryotic dominance in a dataset collected from BML (Aguilar et al. 2016). By contrast, eukaryotic diversity estimated using amplicon sequencing of the V4 region of the 18S rRNA gene detected diverse and sometimes novel eukaryotic microbial diversity (Furgason et al. 2024). Similarly, heteroflagellates were found to be a dominant protist group in BML when the eukaryotic microbial diversity was estimated using V4 (Richardson et al. 2020). A recent study (Furgason et al. 2024) focusing on phytoplankton assessed diversity by 18S rRNA (assessed using V4), 23S rRNA, and 16S rRNA genes (organellar markers), and found that the algal community was diverse and abundant. Notably, however, the euglenid protists (belonging to Discoba) were identified as a highly abundant contributor by 23S and 16S rRNA genes but were nearly undetectable by V4 barcoding of the 18S rRNA gene. Hypervariable regions of the 18S rRNA gene, most commonly either V4 or V9, have been targeted in different studies separately or together to estimate the microbial diversity in different environments (Decelle et al. 2014; de Vargas et al. 2015; Kezlya, Tseplik, and Kulikovskiy 2023; Pagenkopp Lohan et al. 2016; Stoeck et al. 2010), with debate as to the extent to which assays targeting different regions represent accurate pictures of diversity. Both length and primer bias have been raised as possible contributing factors. While the V4 region is located near the middle of the gene and is 247–387 bp in length, the V9 region is located at the 3′‐end of the 18S rRNA gene and is shorter, only 96–134 bp. Moreover, assays targeting the V9 region have been demonstrated to capture some eukaryotic groups (e.g., Metamonada and Discoba) that are missed when targeting the V4 region (Kezlya, Tseplik, and Kulikovskiy 2023; Choi and Park 2020). Thus far the V4 region has been almost exclusively used to assess eukaryotic diversity in oilsands environments, which present unique eukaryotic community compositions.

The present study aims to estimate the relative benefits of using different methods for assessing protist eukaryotic diversity in oilsands environments and determine whether some key groups, such as metamonads or euglenids have been systematically missed in the heavily V4‐based assessments of oilsands environments. Two different DNA regions (V4 vs. V9) were directly compared for characterising the biodiversity in active oilsands tailings ponds (Mildred Lake Settling Basin, MLSB and South‐West‐In Pit, SWIP), a pit lake (BML), and a freshwater reservoir (BCR). Microbial diversity in BML was also assessed using a shotgun metagenomics approach for comparative analysis with amplicon sequencing. The optimal metabarcoding approach depended on the question being asked: each marker provided general community assessment while using both the V4 and V9 hypervariable regions of the 18S rRNA gene may be more informative for identifying the span of eukaryotic diversity.

## 2 Materials and Methods

### 2.1 Sampling, Sample Processing, and Amplicon Sequencing

**FIGURE 1:**
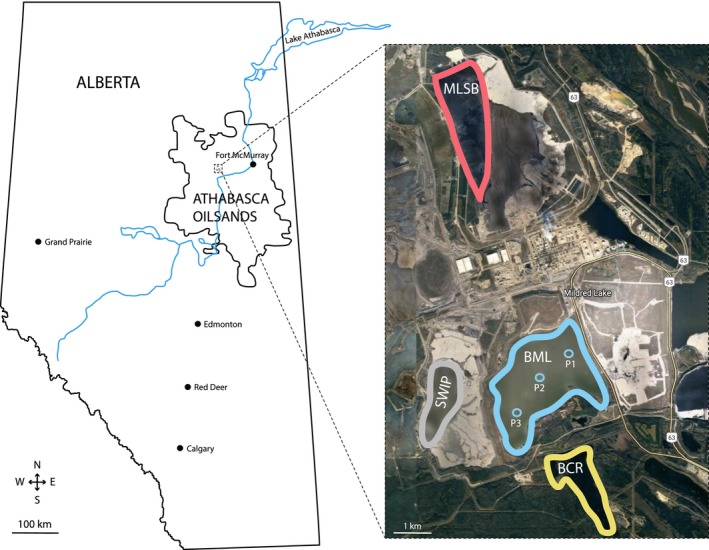
Map of sampling sites. Map of Alberta, situating Base Mine Lake (BML), and expanded view of the inset: BML with three sampling platforms (P1, P2, and P3), Mildred Lake Settling Basin (MLSB), Beaver Creek Reservoir (BCR), and South‐West‐In Pit (SWIP).

Detailed site descriptions and methods for sampling, extraction of microbial DNA, and sequencing analysis of V4‐region amplicons of the 18S rRNA gene have all been described previously (Furgason et al. 2024). Briefly, water from different depths was collected in polycarbonate containers from three sampling platforms (P1, P2, and P3) located in the BML in the months of February, March, July, August, September, and October 2018 as described previously (Albakistani et al. 2022) and in Table S1. Surface water samples were also collected in 2018 from the shorelines of SWIP in August, September, and November, of MLSB in each month from May to September and November, and of BCR each month from May to October. BCR was considered a control for an artificial freshwater reservoir not affected by mining, and MLSB and SWIP for active tailings ponds (Figure 1; Table S1). Water in BML and BCR is oxic year‐round throughout the water column (Saidi‐Mehrabad et al. 2013), while the turbid MLSB waters are suboxic to anoxic because of very high chemical and biological oxygen demands (Saidi‐Mehrabad et al. 2013).

Processing of water samples via centrifugation and extraction of DNA from the pellet material was done as described previously (Albakistani et al. 2022). Primer sequences to amplify the V4 hypervariable region of the 18S rRNA gene were flanked by Illumina adaptors (Table S2). PCR conditions to amplify the V4 region gene fragments were as follows: initial denaturation at 98°C for 3 min; 10 cycles of touchdown PCR, with denaturation at 98°C for 20 s, annealing at 60°C for 30 s (decreasing by 0.5°C per cycle), and extension at 72°C for 30 s, then 30 cycles of denaturation at 98°C for 30 s, annealing at 55°C for 30 s, and extension at 72°C for 30 s; and the final extension at 72°C for 5 min (Stoeck et al. 2010). These data were previously deposited in NCBI under the BioProject accession number PRJNA1003951.

For this study, the V9 hypervariable region of the 18S rRNA gene was amplified (primers provided in Table S2) from the same DNA extracts used to generate V4 amplicons. PCR conditions to amplify the V9 gene fragments were as follows: initial denaturation at 98°C for 3 min; 35 cycles of denaturation at 98°C for 20 s, annealing at 57°C for 30 s, and extension at 72°C for 30 s; and the final extension at 72°C for 7 min. Amplicon libraries were prepared for sequencing using MiSeq Reagent Kit v3 with 175,600 cycles (Illumina part number MS‐102‐3003) and sequenced as described previously (Albakistani et al. 2022). The V9 data were deposited in NCBI under the same BioProject as the V4 data (PRJNA1003951).

### 2.2 OTU Assignment and Phylogenetic Placement

Raw Illumina reads were processed and assigned to operational taxonomical units (OTUs) by ‘Fred's metabarcoding pipeline’ (https://github.com/frederic‐mahe/swarm/wiki/Fred's‐metabarcoding‐pipeline) using Swarm (Mahé et al. 2015) as described previously (Rodríguez‐Martínez et al. 2020) using the V9_DeepSea (Schoenle et al. 2021) and the PR2 (Guillou et al. 2013) databases for V9 and V4 taxonomy assignments, respectively. Possible non‐eukaryotic contamination was removed by blastn v2.10.0+ (Altschul et al. 1990) searches against the NCBI nucleotide database. To mitigate the effect of PCR chimera artefacts, OTUs with total abundances < 10 or those assigned to multicellular organisms (i.e., Metazoa and Embryophyceae) were removed from further analyses.

OTU sequences from the V9 sequencing dataset were mapped onto representative alignments as described previously (Wideman et al. 2020). Briefly, the euglenozoan and metamonad datasets were obtained from EukRef (Kolisko et al. 2020) and enriched with 18S rRNA gene sequences from single‐cell amplified genomes from a recent study (Záhonová et al. 2021) and of barthelonids (Yazaki et al. 2020), *Anaeramoeba* spp. (Stairs et al. 2021; Táborský, Pánek, and Čepička 2017), and free‐living trichomonads (Céza et al. 2022), respectively. OTU sequences were aligned with representative alignments by MAFFT v7.458 (Katoh and Standley 2013) using ‘–addfragments –keeplength’ arguments. The resulting alignment was trimmed using trimAl v1.4.rev15 (Capella‐Gutiérrez, Silla‐Martínez, and Gabaldón 2009) with ‘‐gt 0.25‐cons 50’ parameters. OTU sequences were then removed from the alignment and the reference maximum‐likelihood tree was constructed using RAxML v8.2.12 (Stamatakis 2014) under the GTRCAT model. OTU sequences were then mapped onto the tree using the RAxML evolutionary placement algorithm (EPA) (Berger, Krompass, and Stamatakis 2011) under GTRCAT. Placement signals were accumulated into basal branches using gappa v0.8.4 (Czech, Barbera, and Stamatakis 2020) with threshold 0.8.

### 2.3 Beta Diversity Analyses

For a graphical representation of community dissimilarity, nonmetric multidimensional scaling (NMDS) ordinations were performed in R with the package vegan (Dixon 2003). To identify shared vs. distinct taxa across the V4 and V9 datasets, the OTU tables (Tables [Link], [Link]) were collapsed to the species level. The species‐level composition table was normalised to 6700 counts Scaling with Ranked Subsampling (SRS) (Beule and Karlovsky 2020). Absolute abundances were converted to relative abundance using the function decostand and method total of the package vegan. The Bray–Curtis index was used for the dissimilarity measure with 10,000 iterations. Permutational multivariate analysis of variance (PERMANOVA) (Somerfield, Clarke, and Gorley 2021) was performed in R to assess community composition differences between the V4 and V9 sequencing assays. PERMANOVA was performed using the pairwise.adonis function with the Bray‐Curtis distance method, 10,000 permutations, and *p*‐values adjusted using the Benjamini‐Hochberg method.

### 2.4 Alpha Diversity Analyses

The same OTU data was used for this analysis as for the beta diversity analysis, with Chao1 and Shannon's diversity calculated in *R* using the package otuSummary (Yang 2018). To calculate Faith's phylogenetic diversity (Faith's PD), it is necessary to properly capture microdiversity, therefore this index was calculated based on amplicon sequence variants (ASVs) rather than OTUs. To determine ASVs for calculating Faith's PD, raw sequence data were denoised using the DADA2 algorithm instead of clustered, and then run through Quantitative Insights into Microbial Ecology 2 (QIIME2) version amplicon‐2024.2 (Caporaso et al. 2010) software as described previously (Furgason et al. 2024). Phylogenetic trees were calculated in QIIME2 with FastTree and MAFFT alignment, then those trees were imported into R to calculate Faith's PD with the package Picante (Kembel et al. 2010) and ASV counts normalised to 2500 using the package SRS (Beule and Karlovsky 2020). For the V9 region data, only forward reads were processed due to the short length of the sequence. Fewer ASVs were obtained when the data was processed through QIIME2 compared with the number of OTUs obtained through Swarm, hence the differences in normalisation.

### 2.5 PCR Amplification and Sequencing of Selected Near‐Complete 18S rRNA Genes

Eight OTUs belonging to Euglenozoa and Metamonada groups were selected for further efforts to obtain nearly complete 18S rRNA gene sequences for more accurate phylogenetic identification. V9 amplicon sequences were used to design unique primers targeting each OTU (Table S3). Eukaryotic universal forward and reverse 18S rRNA gene primers (Table S2) were paired with V9 OTU‐specific reverse and forward primers, respectively, for PCR amplification of nearly complete 18S rRNA genes representing each OTU. PCR conditions were as follows: initial denaturation at 98°C for 30 s; 35 cycles of denaturation at 98°C for 10 s, annealing at 62°C (OTU17), 60°C (OTU51), 57°C (OTU46), 61.1°C (OTU140), 58.6°C (OTU193 and OTU40) and 62.1°C (OTU227) for 30 s, extension at 72°C for 1 min and 30 s; and the final extension at 72°C for 5 min. The resulting PCR products were run on a 0.8% agarose gel (Lonza, Rockland, USA), purified with the QIAquick Gel Extraction Kit (QIAGEN GmbH, Hilden, Germany), and sequenced directly in an ABI 3730 automated sequencer (Applied Biosystems) using the specific forward and reverse primers and Standard Sanger DNA sequencing technology. All Sanger sequencing was done at the Molecular Biology Service Unit, University of Alberta, Alberta, Canada.

### 2.6 Metagenome Sequencing, Assembly, and Analysis

Surface (0.60 m for all platforms) and bottom (11.60 m for P1, 13.00 m for P2, and 10.90 m for P3) BML water samples (7.5 L) taken on February 22–26, 2021, were filtered through 8‐μm Nucleopore Track‐Etch membrane filters (Whatman, Kent, UK) to retain eukaryotes on the filter. Subsequently, DNA was isolated using the FastDNA SPIN kit for Soil extraction kit (MP Biomedicals, Santa Ana, USA). Four metagenomes were prepared using NexteraXT DNA Library Prep Kits (Illumina Inc., San Diego, USA), sequenced on partial (20% per metagenome) Illumina MiSeq lanes using MiSeq Reagent Kit v3 (600 cycles) (Illumina Inc.). Subsequently, two selected metagenomes (P1_B and P3_S) were sequenced on two full‐sequencing Illumina MiSeq lanes.

Raw reads were quality‐ and adapter‐trimmed using BBDuk v36.92 (part of BBTools suite: https://jgi.doe.gov/data‐and‐tools/bbtools/). Trimmed reads were assembled using metaSPAdes v3.14.0 (Nurk et al. 2017) in default settings. Qualities of produced assemblies were assessed by QUAST v5.2.0 (Gurevich et al. 2013). Contigs ≥ 1000 bp long in the metagenome assemblies were categorised into eukaryotic, prokaryotic (archaeal and bacterial), organellar (plastidial and mitochondrial) and unclassified sequences by Tiara v1.0.2 (Karlicki, Antonowicz, and Karnkowska 2022). Protein‐coding genes were predicted on identified eukaryotic contigs by MetaEuk v5.34c21f2 (Levy Karin, Mirdita, and Söding 2020) using a reference gene database of marine protists (https://wwwuser.gwdg.de/~compbiol/metaeuk/2019_11/MERC_MMETSP_Uniclust50_profiles.tar.gz). Taxonomy was then assigned by MMseqs2 v13.45111 (Steinegger and Söding 2017) using EukProt v2 (Richter et al. 2022) as a reference database and visualised as Krona plots (Ondov, Bergman, and Phillippy 2011).

### 2.7 Mitochondrial Genome Analyses

Possible mitochondrial contigs were identified in metagenome assemblies by Tiara v1.0.2 (Karlicki, Antonowicz, and Karnkowska 2022) in default settings. Mitochondrial genes were predicted on identified contigs by MFannot (Lang et al. 2023), and their identity was verified by blastn or blastx searches against the NCBI nucleotide or non‐redundant database, respectively.

In the P1_B metagenome, a mitochondrial genome (mtDNA) of *Paramicrosporidium* sp. was identified. To circularise this mtDNA, which contained fragments of a 16S rRNA gene at each end of a linear contig, forward and reverse primers (Table S2) were designed in the 16S rRNA gene. PCR conditions to obtain the full sequence of the 16S rRNA gene were: initial denaturation at 98°C for 30 s; 35 cycles of denaturation at 98°C for 10 s, annealing at 62.5°C for 30 s, extension at 72°C for 1 min and 30 s, and the final extension at 72°C for 5 min. The resulting PCR product was run on a 0.8% agarose gel, purified with the QIAquick Gel Extraction Kit, and sequenced directly in an ABI 3730 automated sequencer using the specific forward and reverse primers. The obtained 205 bp fragment circularised the assembled mtDNA from the metagenome. The protein translations of unknown open reading frames identified by MFannot were subjected to search using Phyre2 (Kelley et al. 2015) for predicting their functions based on remote homology detection. The mtDNA map was drawn by OGDRAW (Greiner, Lehwark, and Bock 2019). Comparison of mtDNAs was done by Mauve aligner using the progressive Mauve algorithm (Darling, Mau, and Perna 2010).

To confirm the identity of the identified mtDNA, the P1_B metagenome was searched for corresponding nuclear 18S rRNA genes and resulted in one matching sequence. A phylogenetic analysis was done using a Fungi‐specific 18S rRNA gene backbone tree (Richardson et al. 2020). Sequences were aligned using MUSCLE v3.8.31 (Edgar 2004). The aligned positions were inspected, masked, and manually trimmed in Mesquite v3.5 (Maddison and Maddison 2023) to include only homologous regions and remove partial or misaligned sequences. Maximum‐likelihood analysis was done in IQTREE v1.6.6 with ultrafast bootstrapping (Minh, Nguyen, and Von Haeseler 2013; Nguyen et al. 2015). Model testing was performed using the built‐in ModelFinder program with the best model selected according to the Bayesian information criterion, and 1000 pseudoreplicates were obtained until tree convergence reached the default convergence coefficient (Kalyaanamoorthy et al. 2017).

## 3 Results

### 3.1 Taxonomic Profiles in BML, BCR and Tailings

To determine the extent to which the V4 and V9 sequencing assays give consistent descriptions of the protist community in oilsands‐associated environments, we PCR‐amplified the V9 regions of extracted DNA from water samples collected in 2018 in four selected sites, that is, BML, MLSB, SWIP, and BCR, and compared the resulting datasets to the PCR‐amplified V4 regions from the same samples previously reported (Furgason et al. 2024). All OTUs assembled are listed in Table S4. After the removal of low‐abundance OTUs and those assigned to multicellular organisms, the remaining total numbers of OTUs were 2987 in the V4 dataset and 1733 in the V9 dataset collectively at all sampling sites. As expected, a very small number of OTUs, represented by 40 and two sequences in the V4 and V9 datasets, respectively, could not be classified as eukaryotes at any taxonomic level, but also failed to retrieve prokaryotic hits (designated ‘No_hit’ in subsequent figures and tables) (Table S4A,B).

**FIGURE 2:**
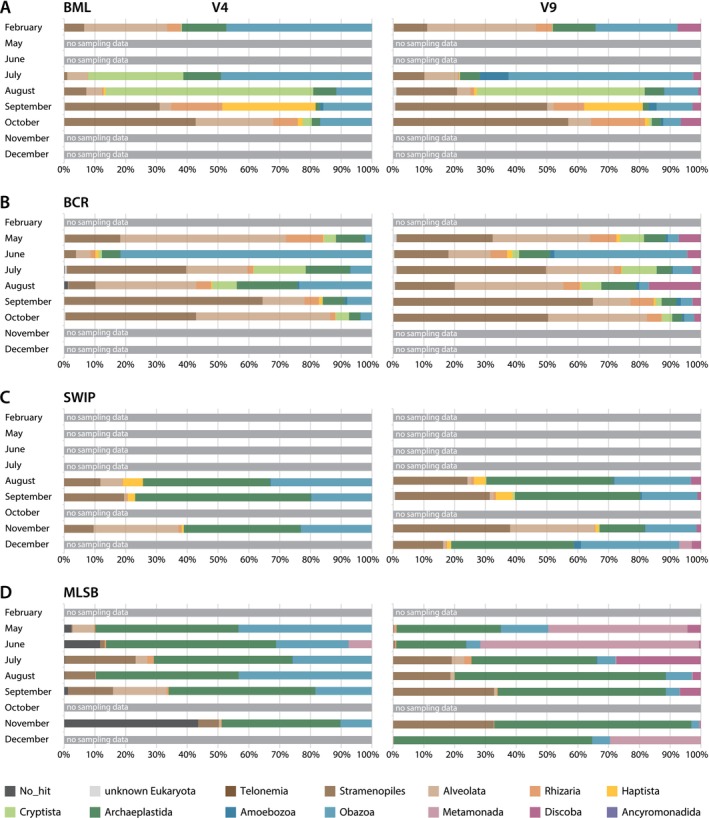
Relative proportion of OTUs related to major eukaryotic groups for each month based on V4 (left) and V9 (right) regions in surface water of sampling sites. (A) BML, (B) BCR, (C) SWIP, (D) MLSB. Eukaryotic groups are colour‐coded according to a figure legend below the graphs.

Consistent with trends from previous reports (Furgason et al. 2024; Richardson et al. 2020), we observed variations in the relative abundances of different eukaryotic (super)groups in different months in all sampling sites in the two datasets, even between the two markers from the same samples. Notably, OTUs assigned to discobans and metamonads were found in high numbers and abundances in the V9 dataset, but rarely found in the V4 dataset (Figure 2; Figure S1). Variations in abundance and identification of species at taxonomic levels below (super)groups were also observed in different months and both V4 and V9 datasets (Table S4K).

In BML, total OTUs identified in the V4 and V9 sequencing datasets were 1259 from 487,704 total reads and 664 from 189,238 total reads, respectively (Figure 2A; Figure S1A; Table S4C,D). Although the communities were dominated by stramenopiles, alveolates, cryptistans, and obazoans in all estimates, their relative abundance estimates were affected by the marker choice. Relative abundances of OTUs belonging to stramenopiles were higher in the V9 dataset (29.24%) compared to the V4 dataset (17.94%) (Table S4K). On the other hand, haptistans and cryptistans showed higher relative abundance in the V4 dataset (9.85% and 25.17%, respectively) than in the V9 dataset (4.19% and 17.77%, respectively). Archaeplastids, alveolates, rhizarians, and obazoans were comparably abundant in both datasets (6.39%, 8.21%, 6.56% and 25.43%, respectively, in the V4 dataset and 5.45%, 8.06%, 5.98% and 22.78%, respectively, in the V9 dataset). Notably, discobans (3.16%) were identified primarily in the V9 dataset (0.02% in the V4 dataset), and metamonads (0.01%) were detected solely in the V9 dataset. Amoebozoans were not detected in the months of February, July and August in the V4 dataset. However, they were found in relatively high relative abundance in the V9 dataset in the same July samples (9.38%).

In BCR, a total of 1618 OTUs from 223,969 total reads and 1363 OTUs from 230,778 total reads were identified by the V4 and V9 sequencing datasets, respectively (Figure 2B; Figure S1B; Table S4E,F). The protist community was highly enriched in stramenopiles (30.50% and 37.37% in the V4 and V9 datasets, respectively), alveolates (18.69% in V4 and 21.88% in V9), and obazoans (30.19% in V4 and 15.72% in V9) (Table S4K). Taxa from the Rhizaria, Cryptista, Archaeplastida, and Discoba were moderately abundant (5%–10% in least one dataset), while all other groups were rare (≤ 1%). Stramenopiles were highly abundant (> 17%) in all months except for June and August in the V4 dataset. In June, obazoans dominated the community (81.67% and 43.21% in the V4 and V9 datasets, respectively). Alveolates were also highly abundant (> 19%) in May, July, August and October in at least one dataset. Discobans were identified by only the V9 sequencing assay and were most abundant (16.79%) in August, while in other months their relative abundance was < 10%. Metamonads were rare in this environment and accounted for < 0.08% of the V9 dataset in all months. Species belonging to ancyromonads and telonemids were found only in BCR samples, however, also very rarely (< 0.1%), with telonemids only detected by the V4 region.

In SWIP, the total OTUs identified by sequencing the V4 and V9 sequencing assays was 748 from 187,040 total reads and 476 from 201,418 total reads, respectively (Figure 2C; Figure S1C; Table S4G,H). SWIP was most dominated by archaeplastids, obazoans, and stramenopiles in both V4 (44.08%, 27.06% and 12.94%, respectively) and V9 datasets (31.67%, 20.70% and 30.14%, respectively) (Table S4K). Similar relative abundances for the V4 (11.26% and 3.98%, respectively) and V9 (10.39% and 3.09%, respectively) datasets were found for alveolates and haptistans. Again, metamonads and discobans were found only in the V9 dataset. Fluctuations of relative abundances were documented in different months (Table S4K). The highest relative abundances of stramenopiles were observed in September (19.39% and 30.80% in the V4 and V9 assays, respectively), which rapidly dropped in November in the V4 dataset (9.59%) and increased to 37.98% in the V9 dataset. Alveolates were abundant only in November in the V4 dataset (27.62%); all other relative abundances were 1%–7%. On the other hand, archaeplastids and obazoans were abundant in all three sampled months in both datasets (above 38% except in November in V4 and 18%, respectively). Only one OTU with a total abundance of 10 was identified as discoban in the V4 dataset in August and September (0.01% and 0.00% (to two significant digits), respectively). However, this OTU could represent a false positive because of the low percent identity (70%) to a reference sequence. On the other hand, 43 OTUs that were assigned to Discoba in the V9 dataset (3.28% and 1.13% in August and September, respectively) had low (66.9%) to the highest percent (100%) identity to a reference.

The biggest difference in the picture of community composition between markers was in MLSB. The total number of OTUs identified in the V4 sequencing dataset was higher (778 from 511,776 total reads) than the V9 sequencing dataset (548 from 394,826 total reads) (Figure 2D; Figure S1D; Table S4I,J). The microbial diversity in MLSB was dominated by archaeplastids (45.08% and 48.15% in the V4 and V9 datasets, respectively), followed by stramenopiles (11.68% and 16.43% in the V4 and V9 datasets, respectively) and obazoans (20.24% in V4 and 5.91% in V9). However, it was in the estimate of metamonads where the major differences occurred (1.08% and 22.01% in the V4 and V9 datasets, respectively). The dynamics of microbial diversity were highly variable in different months. Stramenopiles' relative abundance increased in September and November as compared to May and June. Stramenopiles were higher in relative abundance based on the V9 sequencing assay compared to the V4 assay in September (32.81% vs. 14.60%) and November (32.71% vs. 6.79%). In contrast, alveolates in May (6.99% vs. 0.20%), September (17.40% vs. 0.82%), and November (0.48% vs. 0.08%) and obazoans in all months were more abundant in V4 dataset compared to V9 dataset. OTUs identified as from Rhizaria, Cryptista, Haptista, and Amoebozoa were negligible in all months (< 1%) except for July in the case of Rhizaria (~2%). Archaeplastids were highly abundant in all months in both datasets. Metamonads were not found in May, September, and November using the V4 dataset, however, they were highly abundant in May (45.18%) and June (70.99%) using the V9 dataset. Discobans were not detected in the V4 dataset at all. In contrast, in the V9 dataset, they were identified in all months with peak abundance in July (27.44%).

### 3.2 Taxonomic Profiles in Different Depths of BML, Including Sediment

Differences in protist communities were documented at different depths (2 m, 4 m, 6 m, 8 m, 10 m and bottom) at three platforms (P1, P2, and P3) in BML (Figure 1; Figure S2A; Table S5). Notably ‘bottom’ refers to the deepest sample obtained from each platform and varies in actual depth (Table S5) due to variability in the depth of the consolidated tailings across BML. It does not refer to sediment, which is treated separately below.

The maximum relative abundance of stramenopiles was found at 8 m of depth at P2 and P3 during March in both datasets. Alveolates were found in all depths, however, with lower abundances when the samples were collected from 8 m in March or the deepest sample from each platform in September. The relative abundance of Rhizaria increased with the depth in the water column. Haptista and Cryptista were found in the bottom samples mainly in September and July, respectively. Archaeplastids were most abundant in shallower depths. Amoebozoans were present also mainly in bottom samples, however, in much lower relative abundances. Obazoans were abundant in all samples accounting for > 80% in July at all platforms in the V4 sequencing dataset. Metamonads were detected at negligible abundances in all samples. Discobans were identified in all depths but mainly in the V9 dataset.

Sediment samples were collected in March from under the P1 and P3 platforms in BML (Figure 1; Figure S2B; Table S6). The relative abundances of eukaryotic groups using both the V4 and V9 markers were similar with exceptions already shown in samples from different environments (Figure 2; Figure S2A).

Thus, like the different environments, the V4 and V9 sequencing assays show diverse protistan communities, but with variations in detection and relative abundance of different taxonomic groups by marker. The V9 markers detected higher numbers of metamonads and discobans, whereas the V4 region covered a better representation of obazoans. These effects were most striking in the assessment of ‘bottom’ samples (Figure S2A).

### 3.3 Beta Diversity Analysis Using V4 versus V9 Markers

**FIGURE 3:**
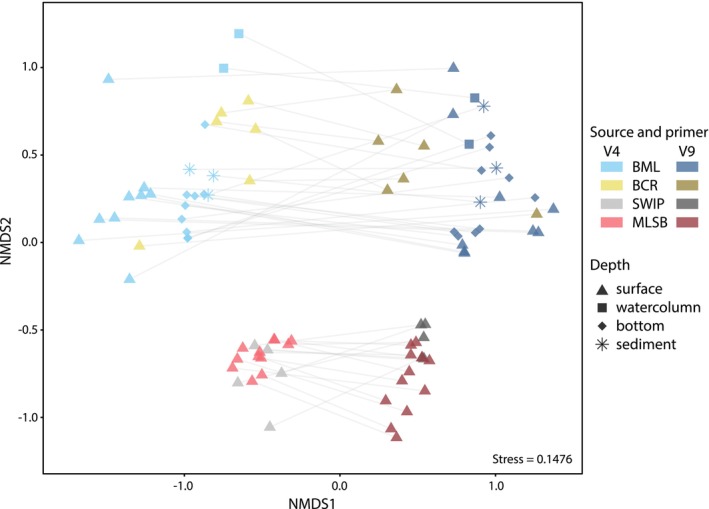
Beta diversity. NMDS plot of BML, BCR, SWIP, and MLSB waters based on Bray–Curtis dissimilarities of eukaryote communities for samples shared between the V4 and V9 region datasets. Data were normalised using scaling with ranked subsampling (SRS) (Beule and Karlovsky 2020) to 6700 counts and taxa were classified at the species level. *k* = 3 axes and the stress score was 0.1476. Symbols are transparent so the overlap of some samples is visible. The graph shows shared samples connected by grey lines.

While the V4 and V9 markers provide different information about which taxa are present in the samples, we wanted to assess whether they gave comparable information about the overall community similarity at the species level across the different environments. Samples from the V4 and V9 sequencing datasets showed distinct clustering of community compositions based on non‐metric multi‐dimensional scaling ordination (NMDS) at the species level (Figure 3). PERMANOVA results indicated that community compositions between the V4 and V9 datasets were significantly different (Table S7). Based on PERMANOVA, MLSB had the most variability explained by differences in the V4 versus V9 sequencing regions (37%).

Notably, despite clear differences between the community compositions of the V4 and V9 sequencing datasets, the same fundamental pattern of environmental clustering was observed within the comparisons by the same marker (Figure S3). That is, in both the V4 and V9 datasets, it was possible to distinguish distinct clusters for the BML/BCR versus the SWIP/MLSB samples.

### 3.4 Alpha Diversity Analysis Using V4 versus V9 Markers

**FIGURE 4:**
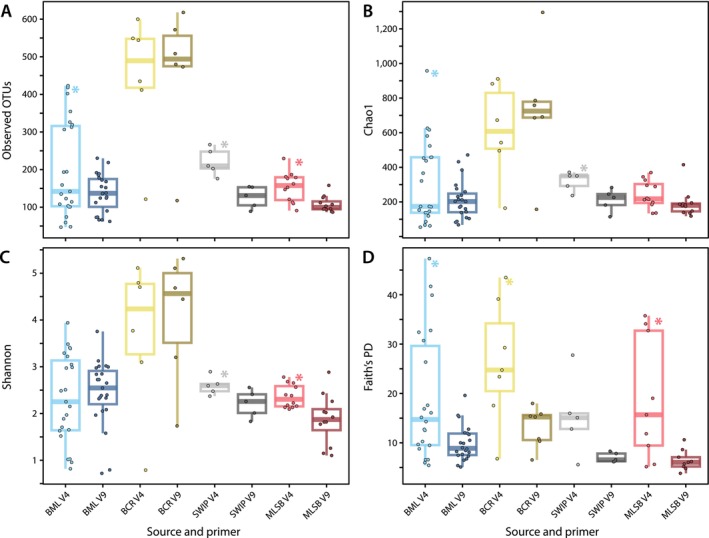
Alpha diversity. Boxplots of four different alpha diversity indices separated by environmental source and by two separate V4‐ and V9‐region sequencing assays. All panels had data normalised to 6700 OTUs using SRS (Beule and Karlovsky 2020), except panel D which had data normalised to 2500 ASVs (see Section 2). Asterisks (*) mark environmental sources that had significantly higher diversity estimated using the V4 assay compared to the V9 assay (*p* < 0.05) based on paired *t*‐tests (see Table S8). No source had significantly higher diversity for the V9 assay compared to the V4 assay.

When samples from all sources were considered together in a paired *t‐*test with normalised reads, the observed number of OTUs, Chao1 and Faith's PD index was significantly higher for the V4 sequencing dataset compared to the V9 dataset (*p* < 0.05) (Figure 4; Table S8). The Shannon index did not vary significantly. When sources were considered independently, most diversity statistics were significantly higher for the V4 dataset versus the V9 dataset, with none significantly higher for the V9 dataset (Table S8).

Faith's PD was also significantly higher in the V4 versus V9 dataset for BML, BCR, and MLSB. Although most of the same phyla were identified by both sequencing assays, the V4 assay identified ~ 1.5 times more ASVs compared to the V9 assay before normalisation (2017 vs. 1324 ASVs, respectively), and generally more taxonomic groups (e.g., for Ciliophora, 152 ASVs with 12 subgroups were found in the V4 dataset vs. 87 ASVs with 8 subgroups in the V9 dataset). Differences in Faith's PD therefore resulted from both higher microdiversity at the ASV level and greater diversity at higher taxonomic levels. Differences in Faith's PD also reflected a larger proportion of unassigned eukaryotic sequences in the V4 sequencing assay compared to the V9 sequencing assay (610 vs. 382 ASVs, respectively).

### 3.5 Precise Phylogenetic Placement of OTUs Found Only in the V9 Sequencing Dataset

The V9 marker revealed discobans and metamonads that had been previously underestimated or even undetected from oilsands environments. To verify and further explore the taxonomic classification of these OTUs, we performed a phylogenetic placement analysis. The vast majority of discoban OTUs belonged to euglenozoans (Table S4). Hence, a specific tree for this group was built (Figure S4). Out of 209 euglenozoan OTUs, 161 belonged to euglenids, 47 to kinetoplastids, and only one to diplonemids. Among euglenids, most OTUs were identified as *Euglena tristella*, *Euglena agilis* , *Euglena pseudostellata,* and members of the genus *Trachelomonas*. Kinetoplastid OTUs belonged mainly to free‐living Metakinetoplastina representatives. However, one OTU mapped on the branch of all parasitic trypanosomatids. The only diplonemid OTU was identified as a *Rhynchopus* species.

The group of metamonads contains many anaerobic protists that often inhabit animal intestinal tracts where they act as endobionts or parasites (Archibald, Simpson, and Slamovits 2017; Muñoz‐Gómez 2023). Nonetheless, the metamonad OTUs in MLSB mapped only onto branches of free‐living species (Figure S5). Among fornicates, all three OTUs mapped on the secondarily free‐living *Trepomonas*. In parabasalids (18 OTUs), most OTUs were found at branches of *Pseudotrichomonas keilini* (13 OTUs) and *Lacusteria cypriaca* (four OTUs). Four OTUs mapped also in preaxostylans, namely on the *Paratrimastix pyriformis* branch.

Since the V9 gene fragment represents a very short portion of the 18S rRNA gene, we aimed to increase the sequence obtained for eight abundant OTUs whose taxonomic placement was poorly resolved by the V9 region alone. Despite repeated attempts and optimization using a forward universal eukaryotic and reverse OTU‐specific V9 designed primers or forward OTU‐specific V9 designed and reverse universal eukaryotic primers (Table S3), we were only successful in extending the gene sequence from two OTUs. We amplified a 423 nt fragment of a euglenid 18S rRNA gene that showed 96.67% identity with *E. tristella*, which was the best blast hit according to E‐value (3e‐120). A 1012 nt long 18S rRNA gene sequence initially identified as a parabasalid, exhibited 99.11% identity with the fornicate *Trepomonas* sp. 3 isolate MACHUPICCHU, a new recently documented strain from Peru (Mazancová et al. 2023).

### 3.6 Metagenomic Profiling and Organellar Genome Assembly

Given the different pictures of the protist community derived from using V4 versus V9 markers and the novel taxa that had been identified in these oilsands‐associated environments, we wanted to assess whether the metagenomic approach might be a fruitful alternative and could yield genomic information informative of cellular adaptations or novelties.

**FIGURE 5:**
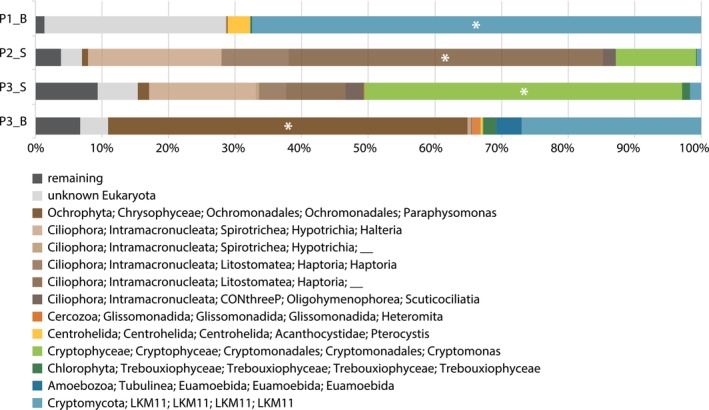
Microbial composition of BML samples for metagenome analysis. The composition at three different sampling platforms (P1‐P3) at surface (S) or bottom (B) was assessed by V4 amplicon sequencing. Enriched taxa from each sample are denoted by white symbols in the corresponding OTU classifications. “Remaining” represents summarised abundance of low abundant and unassigned taxa (neither eukaryotes nor prokaryotes).

To enrich the surface (S) and bottom (B) samples with desired eukaryotes, the samples were size‐filtered. Based on V4 amplicon sequencing performed after the filtering step, all four samples were enriched, in a *Cryptomycota* fungi (P1_B), a ciliate (P2_S), a cryptophyte (P3_S), and a chrysophyte (P3_B) respectively (Figure 5). The metagenome assemblies (Table S9) produced from 400,000 to more than 1000,000 contigs with total length of 253 Mb–1 Gb, and N50 values from 981 to 1595 nt. Further analyses of metagenomes (see Section 2) did not show the prevalence of eukaryotic contigs of the organisms shown as enriched by the V4 analysis. Indeed, it showed a high proportion (45%–57%) of contigs as unclassified in our data (Figure S6) and did not provide any information about the diversity or novel cellular capacities of the protist communities.

**FIGURE 6:**
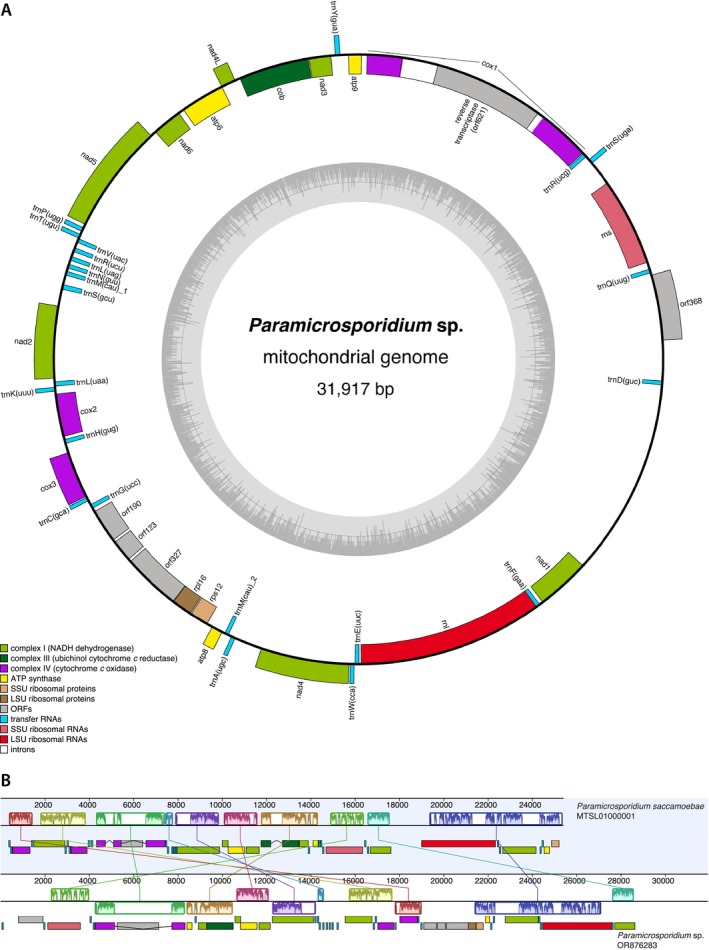
Mitochondrial genome (mtDNA) of *Paramicrosporidium* sp. (A) Map of the *Paramicrosporidium* sp. mtDNA identified in BML. Genes inside and outside the circle are transcribed clockwise and counterclockwise, respectively. The grey circle indicates GC content, 50% threshold is shown by a grey line in the middle. (B) Comparison of the *Paramicrosporidium saccamoebae* and *Paramicrosporidium* sp. mtDNAs. Each coloured region is a locally colinear block (LCB). Below LCBs, the genome as a black line and genes as rectangles coloured as in panel A are shown. LCBs below a genome line (*Paramicrosporidium* sp.) are in the reverse complement orientation relative to the reference genome (*P. saccamoebae*).

Although we were unsuccessful in retrieving genomes of desired protists, we searched the metagenomic assemblies for organellar genomes. To improve the efficacy of the metagenomics approach for identifying microbial diversity, a novel approach has been developed recently based on the assembly of organelles, small‐organelle enriched metagenomics (SoEM) (Jin et al. 2023). In the P1_B metagenome, observed to be enriched in a cryptomycotan OTU (Figure 5), we identified linear mtDNA of a fungus belonging to *Paramicrosporidium* genus, and PCR was performed to circularise this genome (Figure 6A) (no other metagenome assembly contained mtDNA of the enriched taxon). To confirm the presence of *Paramicrosporidium* sp. in this metagenome, we searched for corresponding nuclear 18S rRNA gene sequences and indeed found one. When subjected to a blastn search on NCBI, the best hit was an uncultured fungus (DQ244016.1) with 99% identity. However, the sequence was placed in the clade of microsporidians in the phylogenetic analysis, specifically basal to a clade containing paramicrosporidian species (Figure S7). Therefore, we designated this mitochondrial genome as belonging to *Paramicrosporidium* sp. until microscopic validation can be undertaken.

The only available mtDNA of microsporidians is the circular‐mapping one of *Paramicrosporidium saccamoebae* (Quandt et al. 2017). Although the genes encoded in the *P. saccamoebae* mtDNA were all present in the assembled mtDNA of *Paramicrosporidium* sp., the genomes were not completely colinear but contained nine locally colinear blocks (Figure 6B). While *P. saccamoebae* possesses two and one introns in *cox1* and *cob* genes, respectively, only one and no intron were detected in the *Paramicrosporidium* sp. genes. The *cox1* intron in *P. saccamoebae* encodes LAGLIDADG homing endonuclease, but reverse transcriptase is encoded by this intron in *Paramicrosporidium* sp. In *Paramicrosporidium* sp. mtDNA, the *rpl16* gene was found, but no ribosomal proteins of the large subunit are present in the *P. saccamoebae* mtDNA.

## 4 Discussion

Comparative analyses of microbial communities using different methodologies usually provide a better understanding than any single method. At the same time, large‐scale and longitudinal environmental assessments may need to prioritise simplicity and cost‐effectiveness (Kodikara, Ellul, and Lê Cao 2022). In some cases, the V4 and V9 barcodes are equally useful and tell consistent stories. For example, marine microbial communities assessed by V4 sequencing are similar to those assessed by sequencing complete 18S rRNA genes (Hu et al. 2015; Dunthorn et al. 2012). Nonetheless, many studies have targeted both the V4 and V9 hypervariable regions to more fully elucidate eukaryotic communities (e.g., (Decelle et al. 2014; Dunthorn et al. 2012)). In previous studies, sequencing of the V4 region was used to understand the distribution of microbial eukaryotes in BML (Aguilar et al. 2016). In the present study, we have examined how the V4 and V9 regions capture similar and different stories regarding the microbial eukaryotic communities of oilsands‐associated environments. We also report a metagenomic analysis of eukaryote‐enriched water samples, to assess the utility of this approach to overcome primer biases and obtain genomic information for taxa of interest.

The taxonomic assignment of the protistan communities did differ depending on the region assessed, as expected. The number of unclassified taxa with no eukaryotic hits was higher in the V4 sequencing dataset (40) than in the V9 dataset (2). While there are very few such unclassified OTUs, in the case of the V4 dataset some were quite abundant (e.g., OTU4 had a count of 66,181). Some of the highly abundant OTUs, such as those belonging to *Hexamita* (Metamonada), were identified only by the V9 dataset but remained unclassified by the V4 dataset. This echoes other reports, for example, the V9 sequencing assay identified more diversity than the V4 assay at the community level in tropical lagoon sites of the South Pacific (Kim et al. 2016) and at the Long‐Term Ecological Research station Mare Chiara in the Gulf of Naples (Piredda et al. 2017).

Overall, we found that across all environments, the V4 and V9 markers both showed diverse protist communities. However, each marker was informative about different protist groups and thus a different picture of the dominant taxa in each environment was obtained depending on the marker used. This is particularly relevant when assessing low‐oxygen environments such as tailings. Moreover, sequencing of the V9 region has the potential to reveal different protist taxa than sequencing the V4 region alone (Kezlya, Tseplik, and Kulikovskiy 2023; Choi and Park 2020). This effect is most pronounced and thus relevant when considering tailings vs. fresh‐water environments, and bottom vs. water column samples. Discobans, metamonads, and amoebozoans were largely identified by V9 region only in BML, MLSB, and BCR. *Procryptobia sorokini*, present at low abundance, was the only discoban identified by sequencing the V4 region with more than 99% identity in all samples. Euglenozoa, a class of discobans, was detected by sequencing of the V9 region but not the V4 region, which was expected since primers targeting the V4 region are known for not amplifying ‘excavates’ efficiently (e.g., (del Campo et al. 2019)). Euglenozoans have also been detected before in BML, MLSB, and BCR based on other primer sets and cell count data (Furgason et al. 2024). Metamonads were completely missed in BML and SWIP when the V4 region was sequenced, but notably, in the V9 dataset up to five OTUs were identified in both environments. This also indicates the underestimation of some of the major eukaryotic groups when analysing the V4 region, as noted previously (Choi and Park 2020; Han et al. 2022). The most abundant OTUs in MLSB identified by both V4 and V9 belonged to the supergroup Archaeplastida, more specifically to the green‐algal order Chlorellales. Similarly, in ore mine tailings samples from Southern China, V9‐based OTUs belonging to Archaeplastida were the most abundant, followed by alveolates and stramenopiles (Li et al. 2022). The ratio of phototrophs increased with primary succession in these mine tailings as compared to consumers (Li et al. 2022). The elevated salinity due to high Na^+^, Cl^−^ and HCO^3−^ results in the inhabitation of the salt‐tolerant primary autotrophs and zooplankton such as *Daphnia pulex* (White and Liber 2018). Thus, the importance of which marker to assess depends on the study question. If a study aims to understand and compare the eukaryotic diversity for a more complete picture and functionality of aquatic ecosystems in reclamation sites and tailings ponds, then our data indicates the importance of using both hypervariable regions. In cases where anaerobic protists (such as metamonads) are predicted, e.g., anoxic environments like active tailings ponds, then V9 may be more important to include than environments with a more oxic status.

This difference in the taxonomic composition of the OTUs obtained is reflected to some extent by the community analysis. Distinct clustering between the V4 and V9 datasets was evident in the NMDS plots using the species‐level data. Clustering by BML and BCR vs. the tailings sources, MLSB and SWIP, was evident for both the V4 and V9 sequencing assays, as has been previously demonstrated (Furgason et al. 2024). PERMANOVA results indicated significant differences in community compositions between the V4 and V9 sequencing assays. Based on these data, each marker detects significantly different eukaryotic community compositions, and both would be required to describe a community in more detail, particularly at more precise taxonomic levels (e.g., species‐level). However, based on our results, either marker is suitable for monitoring overall community composition and comparing changes over time or across samples.

Alpha diversity indices were generally higher for the V4 region compared to the V9 region, as significantly higher diversities were reported based on paired *t*‐tests for the V4 region for some sources and indices but not for the V9 region. Thus, while the V4 region notably misses certain taxonomic groups (e.g., metamonads, discobans, and amoebozoans) detected by the V9 region in this study and others (Choi and Park 2020; Piredda et al. 2017), the V4 region captured greater species diversity in this study. This is also reflected in the greater number of OTUs identified by the V4 region compared to the V9 region. In contrast, other studies have reported greater numbers of OTUs detected by the V9 region compared to the V4 region (Choi and Park 2020; Han et al. 2022; Tragin, Zingone, and Vaulot 2018), although higher clustering thresholds (e.g., 99% vs. 97%) tend to result in relatively more OTU diversity assessed via the V4 assay (Stoeck et al. 2010). Our study used Swarm to process sequencing data, which does not cluster OTUs by percent similarity, rather it adds amplicons together iteratively based on a specified small local clustering threshold (Mahé et al. 2015). The differences in diversities could be partly explained by the shorter read length and lesser variability of the V9 region compared to the V4 region (Monier, Worden, and Richards 2016; Tragin and Vaulot 2018). Limitations in reference database sequences for the V9 region also likely play a role in differences in observed OTUs here and other works, as the PR2 database contains ~ 195,000 sequences spanning the V4 region and the V9_DeepSea database contains ~ 37,000 sequences spanning the V9 region (Guillou et al. 2013). Although enriched, the V9_DeepSea database used here adds 18S rRNA gene sequences of only 102 protists (Schoenle et al. 2021). Other studies that compared diversities for each region do not provide a consensus: a study on planktonic protists in the Mediterranean Sea indicated similar Shannon diversities for each marker region (Piredda et al. 2017), while a study on picoeukaryotic communities in the deep sea reported higher Shannon diversity for the V4 region but higher Chao1 diversity for the V9 region (Han et al. 2022).

Because the V9 sequencing analysis identified OTUs that had not previously been observed in oilsands environments, phylogenetic analysis was used to confirm their classification and assess their relationship to known isolates. In many cases, the placement of discoban and metamonad OTUs on the respective phylogenetic trees within the expected clades strengthens their identification. However, some OTUs had less similarity and could represent so far undescribed species.

In addition to amplicon sequencing, we explored the microbial community of BML using a metagenomic approach. Although our samples were filtered to enrich for eukaryotes generally, and for one dominant OTU in each sample before DNA extraction, our sequencing did not allow useful interpretations of the metagenomic data. This may have been due to over‐representation of the prokaryotic community, and thus, excessive background noise within the eukaryotic reads. It may also have been due to the mixed eukaryotic community in the environmental samples and the size and complexity of eukaryotic genomes, including non‐coding DNA and repetitive elements. Although these obstacles could be overcome by a brute‐force sequencing approach, this seems unlikely to be cost‐effective currently. A single‐cell sequencing selecting for organisms of interest may be a more fruitful approach, particularly for genomic information about potential cellular or metabolic adaptations to oilsands environments.

The metagenomic data did nonetheless yield some new information. Analysis with the SoEM approach (Jin et al. 2023) yielded a mitochondrial genome of a species belonging to the *Paramicrosporidium* genus. The mtDNA of *Paramicrosporidium* sp. found here showed a high similarity with the *P. saccamoebae* mtDNA. *P. saccamoebae* possesses a conserved electron transport chain and encodes more genes on its mtDNA as compared to related fungi (Quandt et al. 2017). Given that ours is only the second example of a mitochondrial genome from a microsporidian, it should contribute to a better evolutionary understanding of these highly divergent animal parasites.

## 5 Conclusions

Base Mine Lake, an end‐pit lake, has a developing ecosystem with increasing biodiversity, including rare eukaryotic taxa. The exploration of microbial communities in such environments depends on sample processing methods. For large‐scale monitoring, the V4 and V9 regions of the 18S rRNA gene can assess environmental communities. For detailed taxonomic information, using both markers is necessary to capture the full diversity of protists in these important but underexplored environments.

## Author Contributions

**Kristína Záhonová:** investigation, writing – original draft, validation, visualization, writing – review and editing, data curation. **Harpreet Kaur:** investigation, writing – original draft, validation, visualization, writing – review and editing, data curation. **Chantel C. Furgason:** investigation, visualization, writing – review and editing, data curation. **Angela V. Smirnova:** investigation, data curation. **Peter F. Dunfield:** writing – review and editing, validation, project administration, funding acquisition, supervision, resources. **Joel B. Dacks:** conceptualization, funding acquisition, writing – review and editing, validation, project administration, supervision, resources.

## Conflicts of Interest

The authors declare no conflicts of interest.

## Supporting information


**Figure S1.** Relative proportion of OTUs related to major eukaryotic groups for each sampling day based on V4 (left) and V9 (right) regions in surface water of sampling sites. (A) BML, (B) BCR, (C) SWIP, and (D) MLSB. Eukaryotic groups are colour coded according to a figure legend below the graphs.

**Figure S2.** Relative proportion of OTUs related to major eukaryotic groups based on V4 (left) and V9 (right) regions at (A) different depths of BML platforms P1, P2, and P3 and (B) sediments of BML. Eukaryotic groups are colour coded according to a figure legend below the graphs.

**Figure S3.** NMDS plot of BML, BCR, MLSB, and SWIP waters based on Bray–Curtis dissimilarities of eukaryote communities for samples shared between the V4 (A) and V9 (B) region datasets. Data were normalised using scaling with ranked subsampling (SRS) (Beule and Karlovsky 2020) to 6700 counts and taxa were classified at the OTU level. *k* = 3 axes and the stress scores were 0.1302 for V4 and 0.1300 for V9. Symbols are transparent so the overlap of some samples is visible.

**Figure S4.** Phylogenetic placement of euglenozoan OTUs on a reference tree. The reference tree was constructed by RAxML and euglenozoan OTUs (coloured circles) were placed by EPA.

**Figure S5.** Phylogenetic placement of metamonad OTUs on a reference tree. The reference tree was constructed by RAxML and metamonad OTUs (coloured circles) were placed by EPA.

**Figure S6.** Krona plots of assembled metagenomes. The left and right panels show taxonomy for all and only eukaryotic contigs from (A) P1_B, (B) P2_S, (C) P3_S, and (D) P3_B metagenomes, respectively. Eukaryotic groups are colour coded as in Figure 2. The lowest taxonomic group, into which the organism that the samples were enriched by filtering belonged, is in bold.

**Figure S7.** Phylogenetic analysis of 18S rRNA gene sequences of fungi including *Paramicrosporidium* sp. sequence from metagenome. Ultrafast bootstrap support values are shown when ≥ 80%.

**Table S1.** Sampling sites and sample collection data for microbial community analysis using V4 (A) and V9 (B) regions of 18S rRNA gene.

**Table S2.** Primers used in this study to amplify V4 and V9 regions. Listed is information about PCR primers used for amplicon sequencing, OTU PCR, and circularization of the *Paramicrosporidium* sp. mtDNA.

**Table S3.** OTUs selected for whole 18S rRNA gene amplification. Listed is information about OTUs, used PCR primers, and obtained 18S rRNA gene sequences.

**Table S4.** OTU tables from different environments. The tables were produced by ‘Fred's metabarcoding pipeline’ (see methods) but OTUs with abundance < 10 and those assigned as metazoans or embryophytes were discarded. (A–B) OTUs found in all environments, (C–D) OTUs found in BML samples, (E–F) OTUs found in BCR samples, (G–H) OTUs found in SWIP samples, and (I–J) OTUs found in MLSB samples from V4 (A, C, E, G, I) and V9 (B, D, F, H, J) amplicons. (K) counts and percentages of OTU abundances per month and environment.

**Table S5.** OTU tables from different depths of BML. The tables were produced by ‘Fred's metabarcoding pipeline’ (see methods) but OTUs with abundance < 10 and those assigned as metazoans or embryophytes were discarded. (A) V4 amplicons, (B) V9 amplicons. (C) Counts and percentages of OTU abundances per sample.

**Table S6.** OTU tables from sediments of BML. The tables were produced by ‘Fred's metabarcoding pipeline’ (see methods) but OTUs with abundance < 10 and those assigned as metazoans or embryophytes were discarded. (A) V4 amplicons, (B) V9 amplicons. (C) Counts and percentages of OTU abundances per sample.

**Table S7.** PERMANOVA statistics (R^2^) comparing eukaryotic community composition at the species level for the V4 and V9 regions. Counts were normalised to 6700 using Scaled Rank Subsampling (SRS) (Beule and Karlovsky 2020). Comparisons were made for all sources and for the individual sources. PERMANOVA was run in R using the package vegan and the function pairwise.adonis with 10,000 permutations, the Bray–Curtis distance method, and the Benjamini–Hochberg method for *p*‐value adjustment. The *R* ^2^ value indicates the proportion of variability in community composition explained by the differences in the region used, with greater variability explained as *R* increases. *p* indicates the significance and significant results are indicated by *.

**Table S8.** *t*‐test statistics calculated for Figure 4. For significance level, *** is < 0.001, ** is < 0.01, * is < 0.05, and— is > 0.05. Bolded p‐values are significant in terms of differences in the diversity index between the V4 and V9 regions. Data for observed OTUs, Chao1, and Shannon were normalised to 6700 counts at the OTU level using the package Scaling with Ranked Subsampling (SRS) (Beule and Karlovsky 2020). Data for Faith PD was normalised to 2500 counts at the ASV level using SRS.

**Table S9.** Statistics of metagenome assemblies. P, platform; B, bottom; S, surface.

## Data Availability

The V9 sequencing data is openly accessible through NCBI under BioProject PRJNA1003951: https://www.ncbi.nlm.nih.gov/bioproject/PRJNA1003951. Additionally, raw DNA sequencing reads and metagenome assemblies are available under BioProject PRJNA1045392: https://www.ncbi.nlm.nih.gov/bioproject/PRJNA1045392. The mitochondrial genome of Paramicrosporidium sp. has been deposited in the NCBI GenBank database under accession number OR876283. The figshare link for the Figures [Link], [Link] and Tables [Link], [Link] for the paper https://figshare.com/projects/Assessing_protist_communities_in_oilsands_A_comparative_analysis/230855 .
