# Supplementary material for: Comparative Analysis of Protist Communities in Oilsands Tailings Using Amplicon Sequencing and Metagenomics
Source: Environ Microbiol. 2025 Jan 10;27(1):e70029. doi: 10.1111/1462-2920.70029 (PMC11724239; doi:10.1111/1462-2920.70029)
Supplement: Supplementary file 1 — Figure S1. Relative proportion of OTUs related to major eukaryotic groups for each sampling day based on V4 (left) and V9 (right) regions in surface water of sampling sites. (A) BML, (B) BCR, (C) SWIP, and (D) MLSB. Eukaryotic groups are colour coded according to a figure legend below the graphs. [file EMI-27-e70029-s015.pdf]

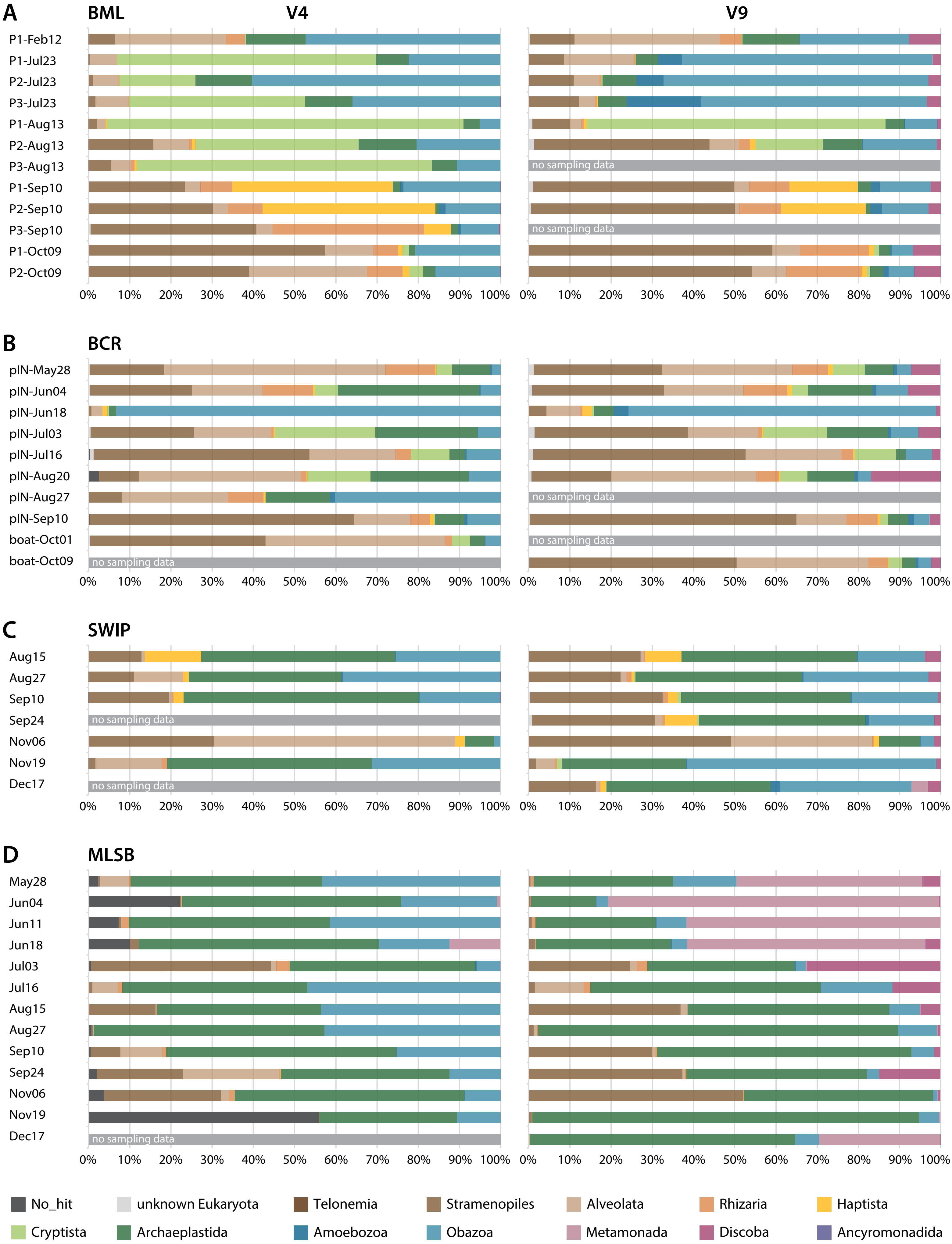
A
BML
V4
V9
P1-Feb12
P1-Jul23
P2-Jul23
P3-Jul23
P1-Aug13
P2-Aug13
P3-Aug13
P1-Sep10
P2-Sep10
P3-Sep10
P1-Oct09
P2-Oct09
no sampling data
0% 10% 20% 30% 40% 50% 60% 70% 80% 90% 100%
B
BCR
pIN-May28
pIN-Jun04
pIN-Jun18
pIN-Jul03
pIN-Jul16
pIN-Aug20
pIN-Aug27
pIN-Sep10
boat-Oct01
boat-Oct09
C
SWIP
Aug15
Aug27
Sep10
Sep24
Nov06
Nov19
Dec17
D
MLSB
May28
Jun04
Jun11
Jun18
Jul03
Jul16
Aug15
Aug27
Sep10
Sep24
Nov06
Nov19
Dec17
No_hit
unknown Eukaryota
Telonemia
Stramenopiles
Alveolata
Rhizaria
Haptista
Cryptista
Archaeplastida
Amoebozoa
Obazoa
Metamonada
Discoba
Ancyromonadida
