# Supplementary figures and images for: Comparative Analysis of Protist Communities in Oilsands Tailings Using Amplicon Sequencing and Metagenomics
Source: Environ Microbiol. 2025 Jan 10;27(1):e70029. doi: 10.1111/1462-2920.70029 (PMC11724239; doi:10.1111/1462-2920.70029)

**A**

**depths**

**V4**

**V9**

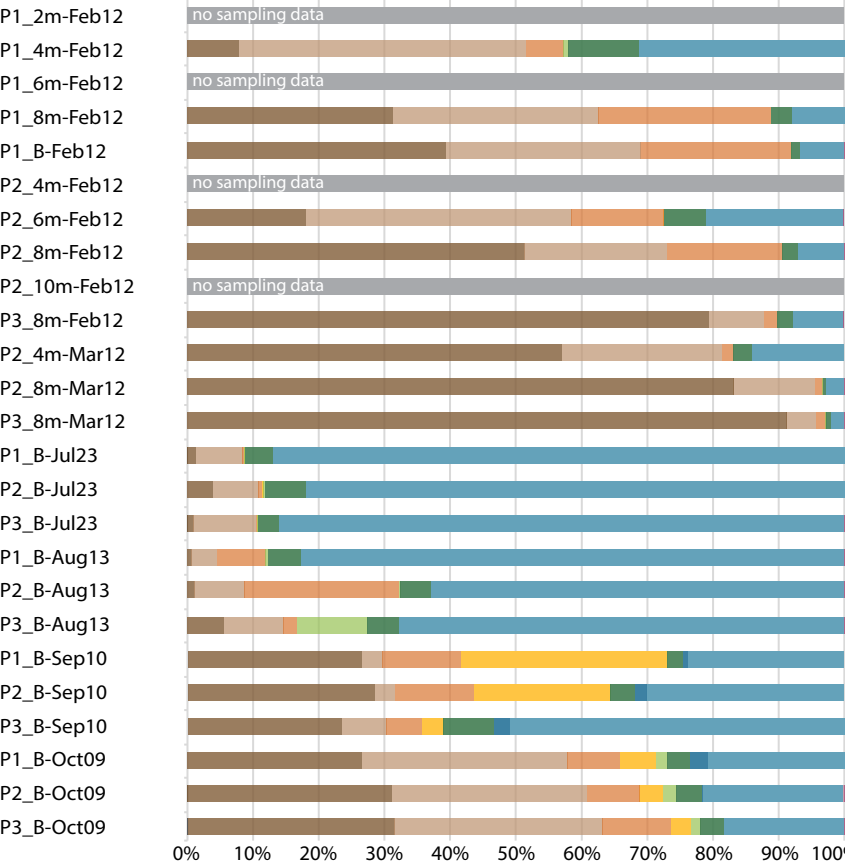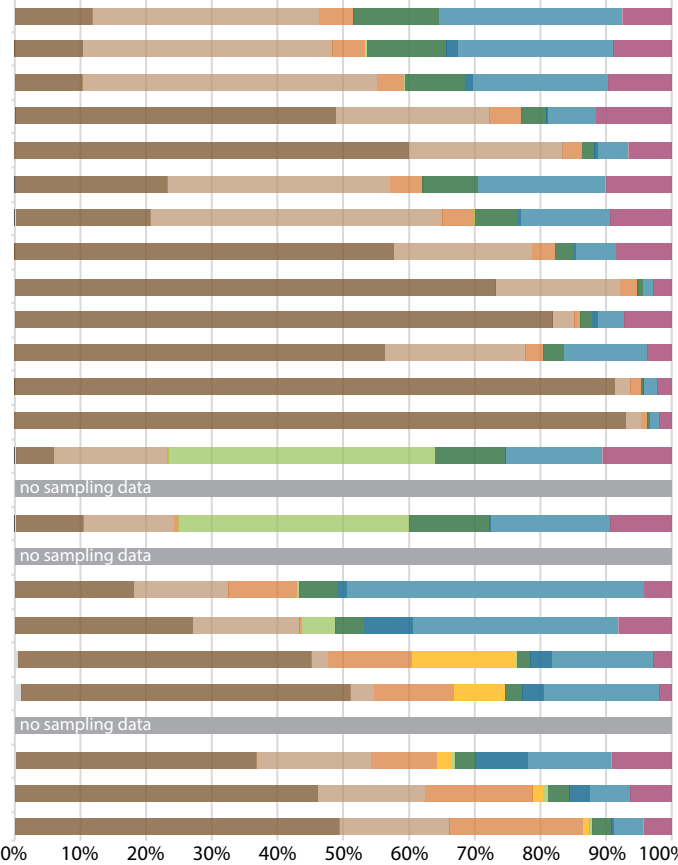

**B**

**sediments**

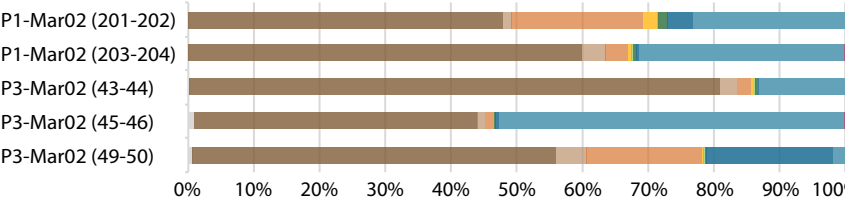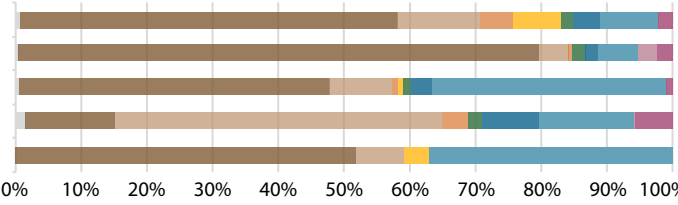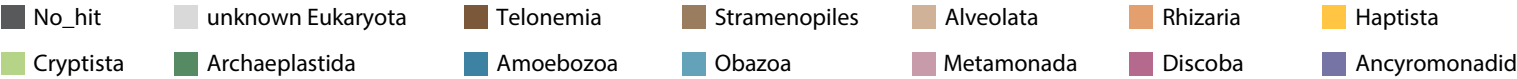

Supplement: Supplementary file 2 — Figure S2. Relative proportion of OTUs related to major eukaryotic groups based on V4 (left) and V9 (right) regions at (A) different depths of BML platforms P1, P2, and P3 and (B) sediments of BML. Eukaryotic groups are colour coded according to a figure legend below the graphs. [file EMI-27-e70029-s013.pdf]

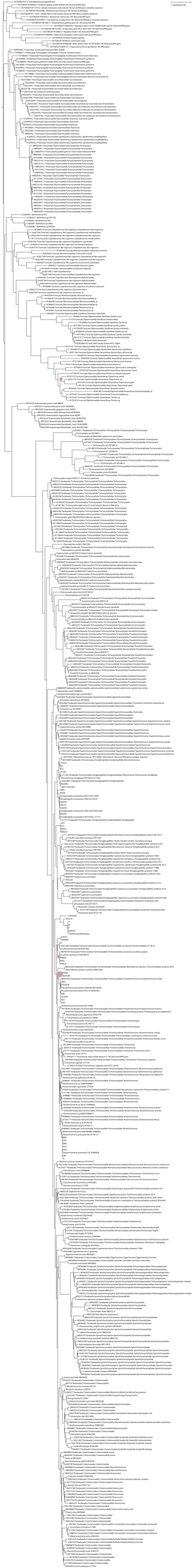

Supplement: Supplementary file 5 — Figure S5. Phylogenetic placement of metamonad OTUs on a reference tree. The reference tree was constructed by RAxML and metamonad OTUs (coloured circles) were placed by EPA. [file EMI-27-e70029-s007.pdf]

A

P1\_B

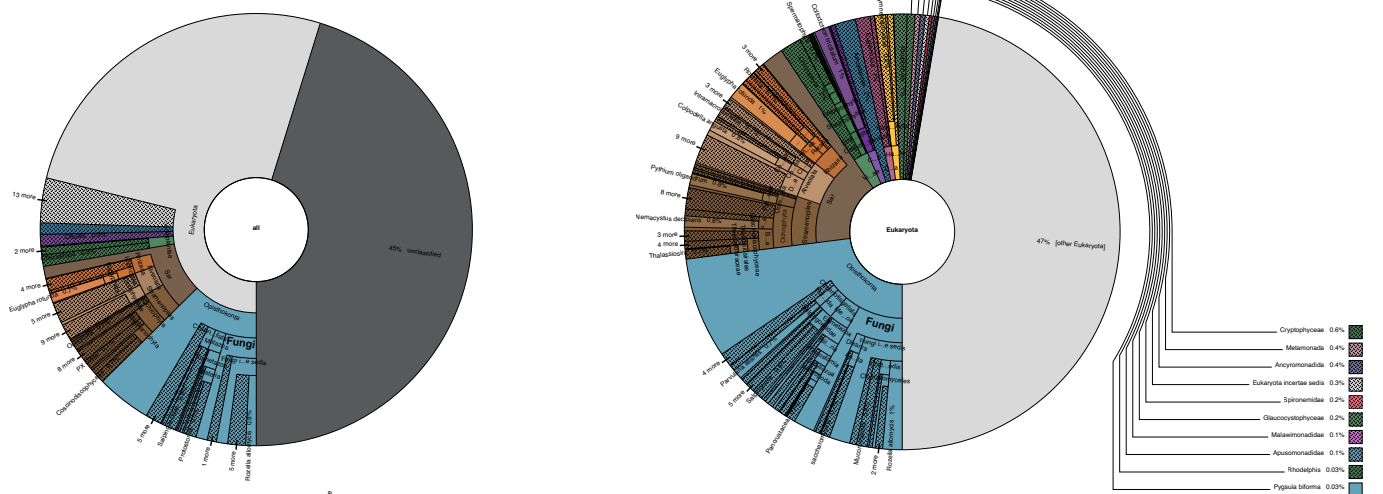

B

P2\_S

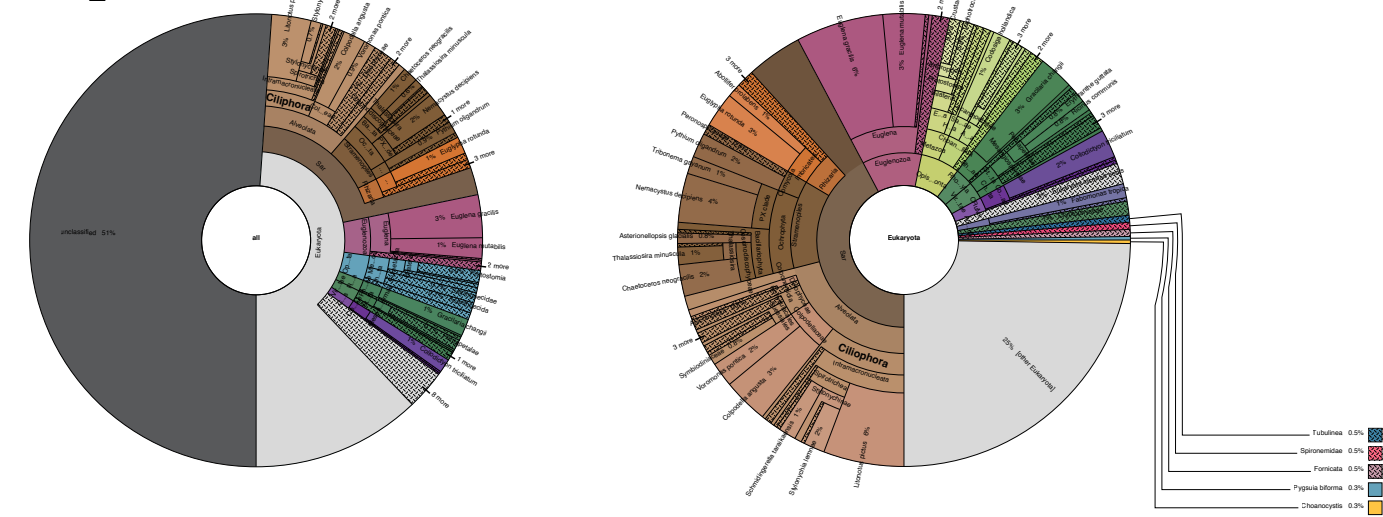

C

P3\_S

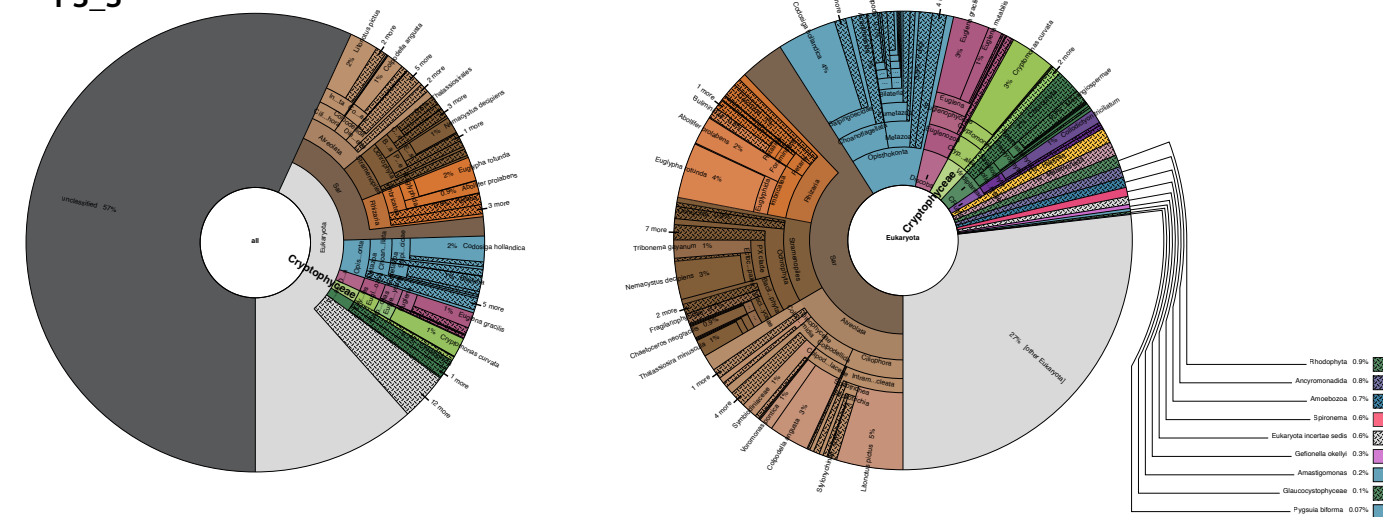

D

P3\_B

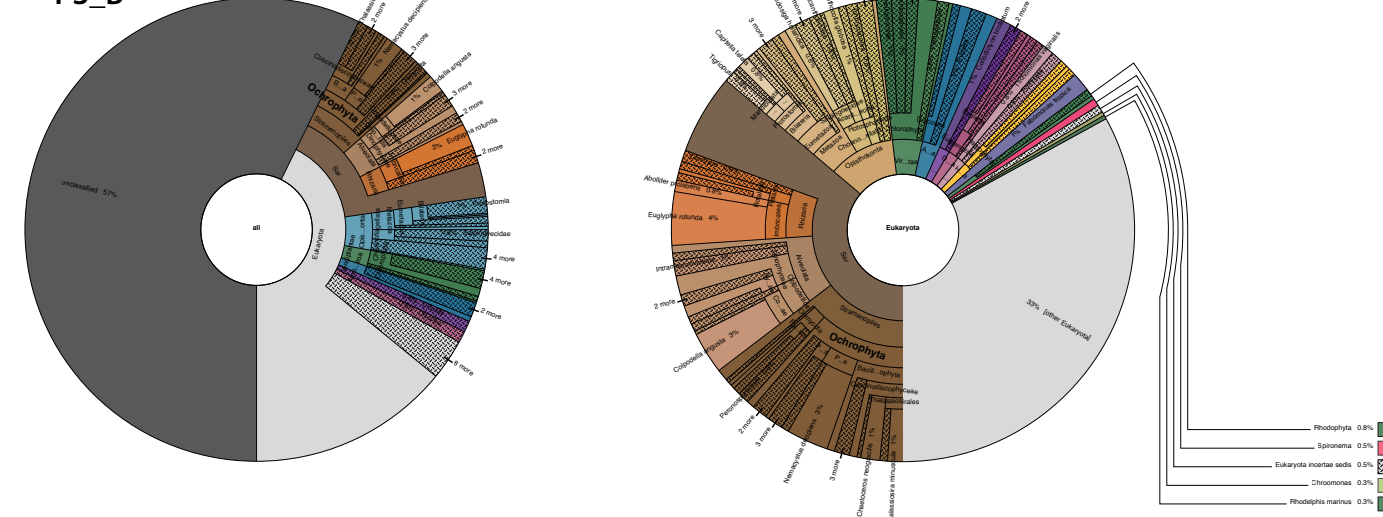

Supplement: Supplementary file 6 — Figure S6. Krona plots of assembled metagenomes. The left and right panels show taxonomy for all and only eukaryotic contigs from (A) P1_B, (B) P2_S, (C) P3_S, and (D) P3_B metagenomes, respectively. Eukaryotic groups are colour coded as in Figure 2. The lowest taxonomic group, into which the organism that the samples were enriched by filtering belonged, is in bold. [file EMI-27-e70029-s011.pdf]

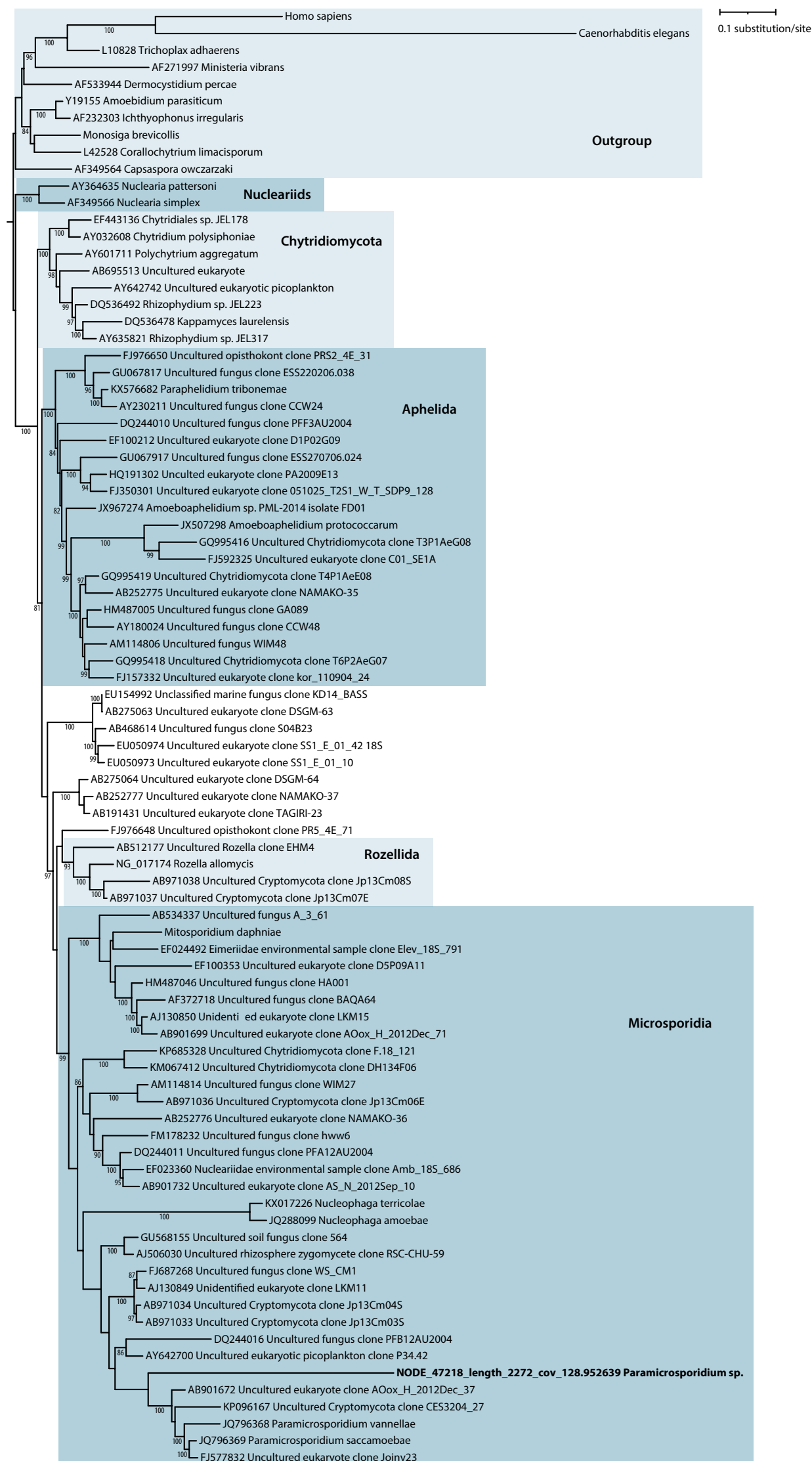

Supplement: Supplementary file 7 — Figure S7. Phylogenetic analysis of 18S rRNA gene sequences of fungi including Paramicrosporidium sp. sequence from metagenome. Ultrafast bootstrap support values are shown when ≥ 80%. [file EMI-27-e70029-s004.pdf]
