# Supplementary material for: Comparative Analysis of Protist Communities in Oilsands Tailings Using Amplicon Sequencing and Metagenomics
Source: Environ Microbiol. 2025 Jan 10;27(1):e70029. doi: 10.1111/1462-2920.70029 (PMC11724239; doi:10.1111/1462-2920.70029)
Supplement: Supplementary file 3 — Figure S3. NMDS plot of BML, BCR, MLSB, and SWIP waters based on Bray–Curtis dissimilarities of eukaryote communities for samples shared between the V4 (A) and V9 (B) region datasets. Data were normalised using scaling with ranked subsampling (SRS) (Beule and Karlovsky 2020) to 6700 counts and taxa were classified at the OTU level. k = 3 axes and the stress scores were 0.1302 for V4 and 0.1300 for V9. Symbols are transparent so the overlap of some samples is visible. [file EMI-27-e70029-s005.pdf]

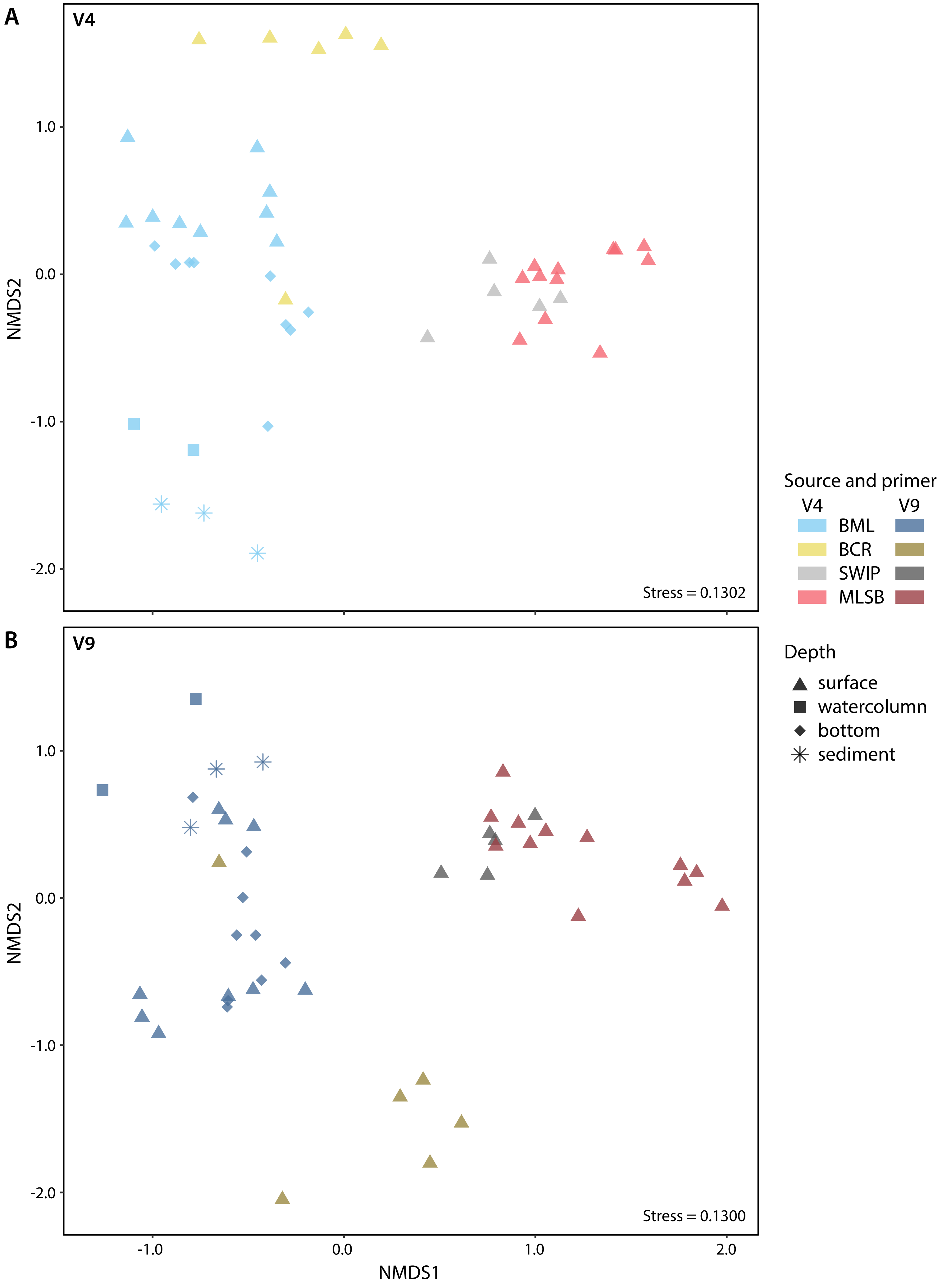
A
V4
1.0
0.0
-1.0
-2.0
NMDS2
Stress = 0.1302
B
V9
1.0
0.0
-1.0
-2.0
NMDS2
Stress = 0.1300
-1.0
0.0
1.0
2.0
NMDS1
Source and primer
V4
V9
BML
BCR
SWIP
MLSB
Depth
surface
watercolumn
bottom
sediment
