# Supplementary material for: Comparative Analysis of Protist Communities in Oilsands Tailings Using Amplicon Sequencing and Metagenomics
Source: Environ Microbiol. 2025 Jan 10;27(1):e70029. doi: 10.1111/1462-2920.70029 (PMC11724239; doi:10.1111/1462-2920.70029)

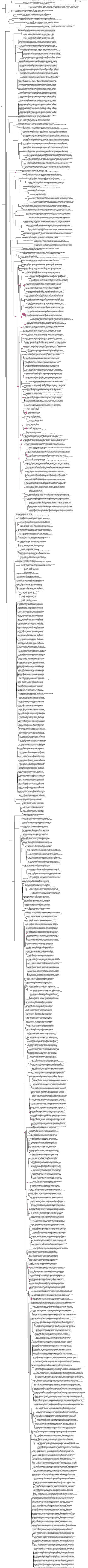

Supplement: Supplementary file 4 — Figure S4. Phylogenetic placement of euglenozoan OTUs on a reference tree. The reference tree was constructed by RAxML and euglenozoan OTUs (coloured circles) were placed by EPA. [file EMI-27-e70029-s001.pdf]
